# Epidemiology of diabetic retinopathy, diabetic macular edema and related vision loss

**DOI:** 10.1186/s40662-015-0026-2

**Published:** 2015-09-30

**Authors:** Ryan Lee, Tien Y. Wong, Charumathi Sabanayagam

**Affiliations:** Singapore Eye Research Institute, Singapore National Eye Centre, Singapore, Singapore; Yong Loo Lin School of Medicine, National University of Singapore, Singapore, Singapore; Ophthalmology and Visual Sciences Academic Clinical Program, Duke-NUS Graduate Medical School, Singapore, Singapore

**Keywords:** Diabetic retinopathy, Diabetic macular edema, Epidemiology, Risk factors

## Abstract

Diabetic retinopathy (DR) is a leading cause of vision-loss globally. Of an estimated 285 million people with diabetes mellitus worldwide, approximately one third have signs of DR and of these, a further one third of DR is vision-threatening DR, including diabetic macular edema (DME). The identification of established modifiable risk factors for DR such as hyperglycemia and hypertension has provided the basis for risk factor control in preventing onset and progression of DR. Additional research investigating novel risk factors has improved our understanding of multiple biological pathways involved in the pathogenesis of DR and DME, especially those involved in inflammation and oxidative stress. Variations in DR prevalence between populations have also sparked interest in genetic studies to identify loci associated with disease susceptibility. In this review, major trends in the prevalence, incidence, progression and regression of DR and DME are explored, and gaps in literature identified. Established and novel risk factors are also extensively reviewed with a focus on landmark studies and updates from the recent literature.

## Introduction

Diabetic Retinopathy (DR) is the leading cause of vision loss in adults aged 20–74 years [1]. From 1990–2010, DR ranked as the fifth most common cause of preventable blindness and fifth most common cause of moderate to severe visual impairment [2]. In 2010, of an estimated 285 million people worldwide with diabetes, over one-third have signs of DR, and a third of these are afflicted with vision-threatening diabetic retinopathy (VTDR), defined as severe non-proliferative DR or proliferative DR (PDR) or the presence of diabetic macular edema (DME) [3]. These estimates are expected to rise further due to the increasing prevalence of diabetes, ageing of the population and increasing of life expectancy of those with diabetes.

PDR is the most common vision-threatening lesion particularly among patients with type 1 diabetes. However, DME is responsible for most of the visual loss experienced by patients with diabetes as it remains the major cause of vision loss in the highly prevalent type 2 diabetes [4] and is invariably present in patients with type 2 diabetes with PDR [5]. In addition to vision loss, DR and DME have also been shown to contribute to the development of other diabetes-related complications including nephropathy, peripheral neuropathy and cardiovascular events [6–9].

The most clinically important risk factors for progression to vision loss include duration of diabetes, hyperglycemia and hypertension. Control of serum glucose and blood pressure have been shown to be effective in preventing vision loss due to DR. Prevalence and risk factors of DR have been studied widely in previous studies including regional and ethnic differences, but epidemiological data on DME are relatively scarce. A review conducted in 2012 suggested that up to 7 % of people with diabetes may have DME and risk factors of DME are largely similar to DR. Recently, new information on the epidemiology of DR and DME has been published from both developed and developing countries. In this review, we summarize the prevalence of DR and highlight regional differences in the epidemiology of DR from recent studies. We also review the incidence, progression and regression of DR and DME, as well as factors contributing to the progression or regression of DR and DME.

## Review

### Prevalence of DR

A pooled individual participant meta-analysis involving 35 studies conducted worldwide from 1980 to 2008, estimated global prevalence of any DR and PDR among patients with diabetes to be 35.4 and 7.5 % respectively [3]. Prevalence of any DR and PDR was higher in those with type 1 diabetes, compared to those with type 2 diabetes (77.3 vs. 25.2 % for any DR, 32.4 vs. 3.0 % for PDR). Table 1 summarizes the findings of various prevalence studies, organized by region, in comparison to the global estimate. Estimates on DR prevalence in type 1 diabetes in Europe and the USA range between 36.5–93.6 %, with VTDR prevalence estimated between 6.7–34.9 % [10–16]. The wide range of prevalence observed may be due to differences in healthcare systems and socioeconomic factors between the studied populations, but conclusions cannot be made as key characteristics such as known duration of diabetes vary greatly between the sampled populations. In the East (Asia and the Middle East), prevalence studies focused on DR in type 2 diabetes alone, due to the low prevalence of type 1 diabetes in these populations. Hence, comparison of DR prevalence between the East and West is restricted only to type 2 diabetes.

**Table 1:** Prevalence of diabetic retinopathy among diabetic subjects

Author (Year)	Type of study	Location	Sample size, Age in years	Diabetes type	Prevalence of DR (%)	Prevalence of VTDR (%)
Yau (2012) [3]	Meta-analysis	Global	12,620	Overall	35.36	11.72 (PDR and/or DME)
			Mean 58.1	Type 1	77.31	38.48 (PDR and/or DME)
			Range 3–97	Type 2	25.16	6.92 (PDR and/or DME)
Asia						
Liu (2012) [22]	Meta-analysis	China	11,996	Unspecified	23.0	2.8 (PDR)
			Range 15–87			
Kung (2014) [19]	Hospital	Hong Kong	15,856	Type 2	12.1	0.3 (PDR)
			Range ≥ 20			
Jee (2013) [202]	Population	South Korea	1678	Type 2	15.8	4.6
			Mean 58.0 ± 11.6			
Wong (2008) [39]	Population	Singapore	757	Type 2	35.0	9.0
			Mean 58.7 ± 11.0			
			Range 40–80			
Chiang (2011) [203]	Population	Singapore	401	Type 2	25.4	Not investigated
			Mean 53.0 ± 9.0			
			Range 40–95			
Zheng (2012) [204]	Population	Singapore	1295	Type 2	30.4	7.1
			Range ≥ 40			
Raman (2009) [21]	Population	India	1,414	Unspecified	18.0	4.3
			Mean 56.1 ± 10.1			
			Range ≥ 40			
Katulanda (2014) [205]	Hospital	Sri Lanka	536	Unspecified	27.4	Not investigated
			Mean 56.4 ± 10.9			
			Range ≥ 18			
Akhter (2013) [31]	Population	Bangladesh	60	Unspecified	21.6	Not investigated
			Mean 46.0 ± 12			
Thapa (2014) [32]	Hospital	Nepal	277	Unspecified	38.26	14.44
			Mean 62.3 ± 13.3			
			Range ≥ 20			
Middle East						
Al-Rubeaan (2015) [64]	Population (Registry)	Saudi Arabia	50,464	Type 2	19.7	10.6 (PDR) 5.7 (DME)
			Mean 59.7 ± 12.8			
			Range ≥ 25			
Al Ghamdi (2012) [26]	Population	Saudi Arabia	612	Unspecified	36.8	17.5 (Scottish DR Grading R4 and/or M2)
			Mean 63.3			
			Range ≥ 50			
Papakonstantinou (2015) [27]	Population	Iran	529	Type 2	29.6	11.1
			Range 40–80			
Europe						
Thomas (2015) [10]	Population	United Kingdom	91,393	Overall	32.4	3.4 (PDR and/or DME)
			Mean 36.5 ± 16.4	Type 1	56.0	11.2 (PDR and/or DME)
			Mean 65.3 ± 11.7	Type 2	30.3	2.9 (PDR and/or DME)
Pugliese (2012) [65]	Hospital	Italy	15,773	Type 2	22.2	9.8
			Range 59–75			
Pedro (2010) [11]	Population	Spain	8675	Overall	26.7	0.59 (PDR)
			Mean 34.9 ± 10.5	Type 1	36.5	1.0 (PDR) 5.73 (DME)
			Mean 64.6 ± 10.8	Type 2	26.1	0.56 (PDR) 6.44 (DME)
Dutra Medeiros (2015) [66]	Population	Portugal	52,739	Type 2	16.3	3.1
			Mean 69.1 ± 11.1			
			Range ≥ 45			
Hautala (2014) [12]	Population	Finland	172	Type 1	93.6	34.9 (PDR)
			Mean 30 ± 3			
			Range 22–35			
Bertelsen (2013) [13]	Population	Norway	514	Overall	26.8	1.2 (PDR) 3.9 (DME)
			Mean 66.4	Type 1	78.0	
			Range 46–87	Type 2	25.0	
Knudsen (2006) [14]	Population	Denmark	984	Overall	48.8	
			Median 37.3	Type 1	53.8	2.9 (PPDR) 5.6 (PDR) 7.9 (CSME)
			IQR 19.0–48.5			
			Median 58.1	Type 2	38.7	3.6 (PPDR) 0.9 (PDR) 12.8 (CSME)
			IQR 15.0–65.0			
Dedov (2009) [15]	Population	Russia	7186	Overall	45.9	8.1 (PPDR) 6.7 (PDR)
			Median 38.0	Type 1	54.6	9.1 (PPDR) 11.1 (PDR)
			IQR 27.0–49.0			
			Median 59.0	Type 2	34.2	7.2 (PPDR) 2.7 (PDR)
			IQR 54.0–66.0			
North America						
Zhang (2010) [18]	Population	United States of America	1006	Unspecified	28.5	4.4
			Range ≥ 40			
Kempen (2004) [17]	Pooled population from 8 studies	United States of America	4440	Type 2	40.3	8.2
			Range ≥ 40			
Roy (2004) [16]	Pooled population from 2 studies	United States of America	1384	Type 1	79.1	31.2
			Range ≥ 18			
Nathoo (2010) [67]	Population	Canada	394	Unspecified	27.2	2.3 (PDR) 2.0 (CSME)
			Mean 58.8			
			Range 10–100			
South America						
Schellini (2014) [206]	Population	Brazil	407	Type 2	7.62	Not investigated
			Mean 51.8 ± 13.6			
			Range ≥ 30			
Esteves (2009) [68]	Hospital	Brazil	437	Type 1	44.4	3.0 (PPDR) 22.2 (PDR) 9.4 (CSME)
			Mean 26.8 ± 7.8			
			Range ≥ 18			
Villena (2011) [69]	Hospital	Peru	1222	Type 2	23.1	1.6 (PPDR) 2.8 (PDR) 2.3 (CSME)
			Median 59.0			
			IQR 52.0–67.0			
Africa						
Thomas (2013) [70]	Hospital	South Africa	5565	Overall	25.8	7.5
			Mean 35.4 ± 15.4	Type 1	36.9	9.7
			Mean 56.8 ± 11.8	Type 2	21.4	6.6
Kahloun (2014) [33]	Hospital	Tunisia	2320	Type 1 and 2	26.3	5.4 (PPDR) 3.4 (PDR) 4.2 (CSME)
			Mean 54.5			
			Range 10–92			
Mathenge (2014) [34]	Population	Kenya	195	Unspecified	35.9	13.9 (PPDR + PDR) 4.1 (CSME)
			Range ≥ 50			
Sharew (2013) [35]	Hospital	Ethiopia	324	Unspecified	41.4	7.3
Oceania						
Kaidonis (2014) [71]	Pooled population from 11 studies	Australia	12,666	Type 1 and 2	30.4	7.5 (PDR and/or DME)
			Range ≥ 15			
Papali’i-Curtin (2013) [207]	Population	New Zealand	5647	Unspecified	19.0	0.4 (PDR)
Win Tin (2014) [208]	Population	Pacific Islands (Vanuatu, Nauru, Solomon Islands)	459	Type 2	47.1	Not investigated
			Mean 54			

*DR* diabetic retinopathy, *VTDR* vision-threatening diabetic retinopathy, *PDR* proliferative diabetic retinopathy, *DME* diabetic macular edema, *PPDR* preproliferative diabetic retinopathy, *CSME* clinically significant macular edema, *IQR* interquartile range

In general, patients with type 2 diabetes in Western communities have a higher prevalence of DR than their Asian counterparts. In the USA, studies estimate that 28.5–40.3 % of patients with type 2 diabetes had DR, and 4.4–8.2 % of them had VTDR [17, 18]. In contrast, most Asian countries report DR prevalence to be between 12.1–23.0 %, and VTDR prevalence to be between 4.3–4.6 % [19–22].

Singapore is a notable exception to this trend. Despite being an Asian country, paralleling the rapid urbanization, industrialization and internal migration that took place over the past five decades in Singapore, DR prevalence in Singapore is reported to be higher (33.9 %) than other Asian countries but comparable to that seen in the Western world [23]. Within the three major ethnic groups in Singapore, the Malays and Indians were reported to have higher a prevalence of DR (33.4 % in Malays, 33.0 % in Indians) compared to the Chinese (25.4 %) [23]. In addition to ethnic differences, a study conducted in Singapore also highlighted geographic heterogeneity in the prevalence of DR within ethnic Indian groups living in Singapore (30.4 %) [24] and in urban India (18 %) [21, 25]. It has been speculated that increased acculturation to a westernized lifestyle associated with increased prevalence of obesity and diabetes, and increased awareness among Indians living in Singapore has led to a higher prevalence, while selective mortality of those with DR in the urban Indian cohorts led to a lower prevalence. In the Middle-East, Saudi Arabia [26] and Iran [27] both report prevalence that are similar to Western communities (36.8 and 29.6 %, respectively). Of concern is that a large proportion of diagnosed DR is vision threatening, with VTDR prevalence estimated to be higher (10.6–17.5 %) than that observed in the Western world. These observations imply that most of these cases of DR have been detected late, when it has already progressed to a vision-threatening stage, or that these populations are particularly susceptible to severe DR due to ethnic predisposition. Other developed Asian countries such as Hong Kong [19] and South Korea [20] report DR prevalence that is much lower than the global average (12.1 and 15.8 %, respectively).

Apart from the east–west divide, rapidly developing economies in Asia such as China and India are observing urban–rural divides in terms of DR disease burden. In China, prevalence of DR was reported to be higher among adults with type 2 diabetes living in rural regions (29.1–43.1 %) [22, 28], compared to their urban counterparts (18.1 %) [22]. Conversely, in a study conducted in Chennai, India, DR prevalence was reported to be higher in urban (18.0 %) [21] compared to rural areas (10.8 %) [29], possibly due to the increasing affluence accompanied by changes in diet in the urban regions and selective mortality of those with diabetes-related complications in rural regions because of poor access to healthcare. The reason why this urban–rural relationship is reversed in China may represent a case of ethnic predisposition, but this is an area that requires further study. In the past two years, reports on DR prevalence from many developing countries in Asia and Africa have been published [30–35]. Prevalence of DR in Sri Lanka, Bangladesh, Nepal, Tunisia, Kenya and Ethiopia ranged from 21.6–41.4 %. While the sample sizes of these studies tend to be smaller, they still provide insight into the burden of DR in these communities.

Although duration of diabetes is a major risk factor for DR, a few studies reported DR prevalence in newly diagnosed diabetes. Prevalence found in these studies ranged from 2.8 % in South Korea to 28.6 % in Singapore [20, 27, 32, 36–39]. Surprisingly, a large percentage (19.2 %) of newly diagnosed patients with diabetes have DR in Scotland, UK, where there is universal healthcare. This prevalence is even higher than in Nepal (13.0 %) [32], where access to healthcare is presumably more limited. However, the prevalence of advanced stages of DR or DME was found to be lower among those with newly diagnosed diabetes suggesting diagnosis of DR early in the course of the disease [40].

### Incidence of DR

There are few population-based cohort studies, outside of the USA or UK, which have investigated DR incidence. Various cohort studies investigating DR incidence over the past two decades are listed in Table 2. Comparisons between the East and West, urban and rural populations, and developed versus developing countries are not possible due to the lack of population-based cohort studies in Asia and many developing countries. In the USA, the Wisconsin Epidemiologic Study of Diabetic Retinopathy (WESDR) found that among patients with insulin-dependent diabetes with onset before the age of 30, who are presumed to have type 1 diabetes, the 4-year cumulative incidence of DR was 59.0 % [41]. At 10, 14 and 25 years, cumulative incidence of DR in the same cohort rose to 89.3 % [42], 95.9 % [43], and 97 % [44], respectively. Similar observations were made in the Danish Cohort of Pediatric Diabetes 1987 (DCPD1987), which reported a 16-year cumulative incidence of 95.1 % [45]. While these cohorts have long follow-up times, it should be noted that the participants were recruited between 1979 and 1989. The incidence reported in these studies may not reflect actual DR incidence today, owing to significant advancements in retinopathy diagnosis techniques and risk factor management in the past three decades. For example, in a UK cohort recruited between 1991 and 1999, 6-year cumulative incidence of DR in patients with type 1 diabetes was estimated to be only 45.3 % [46]. A separate UK study, involving only newly diagnosed cases of type 1 diabetes recruited between 2000 and 2007, found 9-year cumulative incidence of DR to be only 23.9 % [47]. In Finland, the incidence of VTDR was reported to be decreasing in patients with type 1 diabetes [48]. In this study, patients who were diagnosed with diabetes from 1980 to 1984 had 47 % reduced risk of VTDR as compared to patients diagnosed from 1975 to 1979, after adjusting for gender and age of diabetes onset. The reduction in risk was even greater in the cohort diagnosed from 1985 onwards, at 64 %. Overall, these studies indicate that while almost all patients with type 1 diabetes may eventually develop DR over time, the incidence of DR and VTDR among patients with type 1 diabetes is probably on the decline.

**Table 2:** Incidence of diabetic retinopathy among diabetic subjects

Author (Year)	Type of study	Location	Sample size, Age in years	Diabetes type	Follow-up time in years	Cumulative incidence of DR, % (95 % CI)	Incidence of VTDR (%)
Asia							
Xu (2014) [75]	Population	China	2602	Unspecified	10	4.2 (3.45–5.03)**	Not investigated
			Mean 64.6 ± 9.7				
Jin (2014) [98]	Population	China	322	Type 2	5	46.9	13.9 (Severe NPDR)
			Mean 66.1 ± 13.2				4.6 (PDR)
Tam (2009) [60]	Hospital	Hong Kong	212	Type 2	4	20.3	0.47 (PDR)
			Mean 55.2 ± 9.5				
Song (2011) [61]	Hospital	Hong Kong	3647	Type 2	4	15.2	0.03
			Mean 62.60 ± 9.58				
Kawasaki (2011) [62]	Hospital	Japan	1221	Type 2	8	26.6	Not investigated
			Mean 58.2 ± 6.9				
Kajiwara (2014) [76]	Hospital	Japan	383	Type 2	5.8 ± 2.5 (retrospective)	58.5/1000 person years	Not investigated
			Mean 59.4 ± 11.0				
Tsugawa (2012) [209]	Hospital	Japan	1083	Unspecified	3	15.7	Not investigated
			Mean 51.0 ± 11.7				
Ahmed (2012) [210]	Hospital	Bangladesh	977	Type 2	15	50.6 (47.5–53.8)	Not investigated
			Mean 41 ± 8				
Middle East							
Manaviat (2008) [211]	Hospital	Iran	120	Type 2	4	47.5 (38.6–56.4)	Not investigated
			Mean 55.2 ± 9.6				
Janghorbani (2003) [212]	Hospital	Iran	549	Type 2	5.1 ± 2.1 (retrospective)	45.4	Not investigated
			Mean				
			45.7 ± 9.3				
Europe							
Stratton (2001) [51]	Population	United Kingdom	1216	Type 2	6	41	Not investigated
			Mean 52.2 ± 8.5				
Younis (2003) [46]	Population	United Kingdom	305	Type 1	6	45.3 (36.9–53.7)	5.4 (2.3–8.5)
			Median 30.2				
			IQR 21.5–39.8				
Younis (2003) [50]	Population	United Kingdom	3743	Type 2	6	38.1 (35.1–41.2)	6.1 (4.4–7.8)
			Median 63.4				
			IQR 56.1–69.8				
Jones (2012) [52]	Population	United Kingdom	16,444	Type 2	10	66	18.7
			Median 66.7				
			IQR 58.0–74.5				
Martin-Merino (2012) [47]	Population	United Kingdom	1757	Type 1	9	23.9	4.4 (DME)
			Mean 19.1				
			63,226	Type 2		27.8	3.6 (DME)
			Mean 61.3				
Thomas (2012) [49]	Population	United Kingdom	49,763	Type 2	4	26.0	0.7
			Mean 60.2 ± 11.3				
Perol (2012) [213]	Hospital	France	236	Unspecified	3	14.0 (9.5–18.4)	0
			Mean 54.0 ± 12.8				
Romero-Aroca (2011) [77]	Hospital	Spain	334	Type 1	10	35.9	11.07 (DME)
			Mean 25.7 ± 11.7				
Salinero-Fort (2013) [59]	Population	Spain	2405	Type 2	4	8.07 (7.04–9.22)	2.8 (PDR) 1.2 (DME)
			Mean 67.5 ± 10.6				
Henricsson (2003) [111]	Population	Sweden	627	Types 1 and 2	10	39	1.8 (PDR)
			Mean 35.3 ± 5.8				
Broe (2014) [45]	Population	Denmark	185	Type 1	16	95.1	Not investigated
			Mean 7.5 ± 3.7				
North America							
Lee (1992) [55]	Population (Oklahoma Indians)	United States of America	380	Type 2	14	72.3	15.4 (PDR)
			Mean 48.2 ± 8.4			12.5 improved	
Klein (1998) [43]	Population	United States of America	634	Type 1	14	95.9 (93.2–98.6)	26.1 (22.6–29.6) (DME)
			Mean 14.2 ± 7.4				
Tudor (1998) [53]	Population	United States of America	169	Type 2	4	22.5	Not investigated
			Mean 58.1				
Varma (2010) [54]	Population (Latino)	United States of America	775	Unspecified	4	34.0 (30.0–38.0)	5.4 (3.8–7.1) (DME)
			Mean 58 ± 9.7				
Oceania							
Cikamatana (2007) [56]	Population	Australia	150	Unspecified	5	22.2 (14.1–32.2)	Not investigated
			Mean 66.2 ± 8.4				
Others							
Leske (2006) [57]	Population	Barbados	436	Types 1 and 2	9	39.6 (33.6–45.5)	8.3
			Mean 57.6 ± 9.4				
Tapp (2006) [58]	Population	Mauritius	227	Unspecified	6	23.8	0.4 (PDR)
			Mean 50 ± 11				

*DR* diabetic retinopathy, *VTDR* vision-threatening diabetic retinopathy, *CI* confidence interval, *NPDR* nonproliferative diabetic retinopathy, *PDR* proliferative diabetic retinopathy, *DME* diabetic macular edema

**Cumulative incidence of DR among total sample, incidence among participants with diabetes not reported

In the UK, population studies involving patients with type 2 diabetes estimated cumulative incidence of DR to be 26.0 % at 4 years [49] 38.1–41.0 % at 6 years [50, 51], and 66 % at 10 years [52]. These findings seem comparable to that found in US population studies, which estimated cumulative incidence of DR to be 22.5–34.0 % at 4 years [53, 54] and 72.3 % at 14 years [55], despite differences in ethnicity and age of the cohorts at the time of diabetes diagnosis. Cohorts in Australia [56], Barbados [57] and Mauritius [58] report cumulative incidence that is similar to the UK and US studies. In contrast, the 4-year cumulative incidence of DR in a Spanish cohort is much lower, estimated at 8.1 % [59]. Age and duration of diabetes are comparable between the US, UK and Spanish studies, and this significant difference in incidence is attributed to unusually good glycemic control within the Spanish cohort, with mean HbA1c at 7 %, with 55 % of the cohort achieving HbA1c of less than 7 %. In contrast, patients in one of the US cohorts [53] had HbA1c of 9.9 % on average.

As with prevalence, incidence data from Asia is restricted only to that of type 2 diabetes. A population-based study in urban Shanghai, China, found the 5-year cumulative incidence to be much higher than in the US and UK, at 46.9 %, of which more than a third of it is VTDR. This may just be due to differences in known diabetes duration of the cohorts; the Chinese cohort has diabetes duration of 11 years on average at baseline assessment, while studies in the US and UK report diabetes duration to be 4 to 7 years on average. More prospective studies are warranted to compare the incidence of DR in Asia with that observed in Europe or the US.

### Progression and regression of DR

A large number of cohort studies have investigated progression and regression of DR [44, 45, 52–54, 56–58, 60–62]. Disease severity was most often classified by the Early Treatment Diabetic Retinopathy Study (ETDRS) classification for DR severity [63]. The cohort with the longest follow-up time was the WESDR cohort, which reported 25-year progression of DR in patients with type 1 diabetes [44]. In this study, DR severity was assigned a level by concatenating the severity grade in both eyes, with the worse eye given greater weight. This created a 15-step scale, and progression was defined as increase in severity of 2 steps or more. Some other studies assigned DR severity based on the severity grade in the worse eye alone. The findings on DR progression and regression from the various cohort studies are summarized in Table 3. Four to six-year cumulative incidence of 2-step progression among the studies ranged from 24.1 to 38.9 %, which increased to 64.1 and 83.1 % in studies with 16-year or 25-year follow-up.

**Table 3:** Progression and regression of diabetic retinopathy

Author (Year)	Method of severity grading	Progression intervals	Criteria for progression or regression	Progression of DR (%)	Progression from NPDR to PDR (%)	Regression of DR (%)
Asia						
Tam (2009) [60]	Concatenated ETDRS severity of both eyes, with 11 levels	Cumulative at 4 years	2-step	34.7	9.9	13.2
Song (2011) [61]	Eye with worse ETDRS severity	Cumulative at 4 years	1-step	6.6	Not investigated	45.8
Kawasaki (2011) [62]	Mild DR to at least severe NPDR according to ETDRS	Cumulative at 8 years	N/A	N/A	15.9	Not investigated
Europe						
Jones (2012) [52]	Eye with worse ETDRS severity	Cumulative at 5 years	Not stated	Data irretrievable	6.1	Not investigated
		Cumulative at 10 years			9.6	
Broe (2014) [45]	Eye with worse ETDRS severity	Cumulative at 16 years	2-step	64.1	31.0	0
North America						
Tudor (1998) [53]	Eye with worse ETDRS severity	Cumulative at 4 years	2-step	24.1	Not investigated	13.3
Varma (2010) [54]	Concatenated ETDRS severity of both eyes, with 15 levels	Cumulative at 4 years	2-step	38.9	5.3	14.0
Klein (2008) [44]	Concatenated ETDRS severity of both eyes, with 15 levels	Between 0 to 4 years	2-step	13.5 annually	2.5 annually	3.0 annually
		Between 4 to 10 years		13.0 annually	4.0 annually	0.8 annually
		Between 10 to 14 years		12.0 annually	2.5 annually	0.4 annually
		Between 14 to 25 years		2.4 annually	1.5 annually	0.4 annually
		Cumulative at 25 years		83.1	42.0	17.8
Oceania						
Cikamatana (2007) [56]	Concatenated ETDRS severity of both eyes, with 15 levels	Cumulative at 5 years	1-step	25.9	4.1	Not reported
Others						
Leske (2006) [57]	Mild or moderate DR to at least severe NPDR according to ETDRS	Cumulative at 9 years	N/A	N/A	8.2	Not investigated
Tapp (2006) [58]	Mild or moderate DR to at least severe NPDR according to ETDRS	Cumulative at 6 years	N/A	27.7	5.2	Not investigated

*DR* diabetic retinopathy, *NPDR* nonproliferative diabetic retinopathy, *PDR* proliferative diabetic retinopathy, *ETDRS* Early Treatment for Diabetic Retinopathy Study, *N/A* not available

In general, progression was much more common than regression. Two Asian cohort studies, both hospital-based and carried out in Hong Kong, investigated the regression of DR. One of the studies found 4-year progression of DR to be 34.7 % and 4-year regression to be 13.2 % [60], which is similar to that seen in the population-based US cohorts. However, the other study found 4-year regression to be substantially higher (45.8 %) and progression to be lower (6.6 %) [61]. This study defined progression or regression by 1-step change in severity, while most of the other studies defined progression or regression by 2-step changes in severity. Moreover, this study was based in a community optometry clinic. Hence, the population sample may be biased towards patients with mild baseline severity of DR, as patients with more severe disease may have been referred to tertiary hospitals for follow-up. Indeed, 91.7 % of patients with DR at baseline in this study had only mild NPDR, and the 1-step regression of mild NPDR to no DR accounted for the majority of the regression observed in this study. The results of this study are hence not directly comparable with that of the other cohorts, but it highlights the high probability of disease regression in patients with only mild NPDR. The absence of data on population-based cohorts in Asia also precludes direct comparison of progression and regression rates between Asian and Western populations.

### Prevalence of DME

In most studies, DME was defined by hard exudates in the presence of microaneurysms and blot hemorrhages within one disc diameter of the foveal center. Clinically significant macular edema (CSME) is the more severe spectrum of DME, and was defined by the presence of edema within 500 μm of the foveal center, or focal photocoagulation scars present in the macular area. The prevalence of DME among recent cross-sectional studies is summarized in Table 4. Among the population-based studies, prevalence of DME among patients with type 1 diabetes was between 4.2 and 7.9 %. In patients with type 2 diabetes, it was between 1.4 and 12.8 %. Non-stereoscopic fundus photography was used in most studies, which affects the accuracy of DME assessment. About half of the studies defined macular edema using the CSME criteria, and hence only the more severe spectrum of DME was captured in these studies. Overall, the heterogeneity in methodology causes comparison of prevalence between these studies to be a challenge. The prevalence of DME among patients with diabetes is generally much lower than that of DR [11, 13, 14, 16–18, 20, 21, 24, 26, 27, 32–35, 39, 64–71]. There was no observable difference between prevalence of DME between Western or Eastern populations.

**Table 4:** Prevalence of diabetic macular edema among diabetic subjects

Author (Year)	Type of study	Location	Type of diabetes	Prevalence (%)	Definition of macular edema
Yau (2012) [3]	Meta-analysis	Global	Overall	7.48	DME and/or CSME
			Type 1	14.25	DME and/or CSME
			Type 2	5.57	DME and/or CSME
Xie (2008) [214]	Population	China	Unspecified	4	CSME
Jee (2013) [20]	Population	South Korea	Type 2	2.8	DME
				1.4	CSME
Wong (2008) [39]	Population	Singapore	Type 2	5.7	DME
				3.0	CSME
Zheng (2012) [24]	Population	Singapore	Type 2	7.2	DME
				4.5	CSME
Raman (2009) [21]	Population	India	Unspecified	1.4	CSME
Thapa (2014) [32]	Hospital	Nepal	Unspecified	5.78	CSME
Al-Rubeaan (2015) [64]	Population (Registry)	Saudi Arabia	Type 2	5.7	DME
Al Ghamdi (2012) [26]	Population	Saudi Arabia	Unspecified	20.3	Scottish DR Grading M1
				15.9	Scottish DR Grading M2 (M2 equivalent to DME)
Papakonstantinou (2015) [27]	Population	Iran	Type 2	4.7	CSME
Thomas (2015) [10]	Population	United Kingdom	Type 1	4.2	DME
			Type 2	1.4	DME
Pugliese (2012) [65]	Hospital	Italy	Type 2	1.3	DME
Pedro (2010) [11]	Population	Spain	Type 1	5.73	CSME
			Type 2	6.44	CSME
Dutra Medeiros (2015) [66]	Population	Portugal	Type 2	1.4	DME
Bertelsen (2013) [13]	Population	Norway	Types 1 and 2	3.9	DME
Knudsen (2006) [14]	Population	Denmark	Type 1	7.9	CSME
			Type 2	12.8	CSME
Zhang (2010) [18]	Population	United States of America	Unspecified	2.7	CSME
Varma (2014) [215]	Population	United States of America	Unspecified	3.8	DME
Petrella (2012) [216]	Population (registry)	Canada	Type 1 and 2	15.7	DME
Nathoo (2010) [67]	Population	Canada	Unspecified	2.0	CSME
Esteves (2009) [68]	Hospital	Brazil	Type 1	9.4	CSME
Villena (2011) [69]	Hospital	Peru	Type 2	2.3	CSME
Thomas (2013) [70]	Hospital	South Africa	Type 1 and 2	3.2	DME
Kahloun (2014) [33]	Hospital	Tunisia	Type 1 and 2	8.7	DME
				4.2	CSME
Mathenge (2014) [34]	Population	Kenya	Unspecified	33.3	DME
				4.1	CSME
Sharew (2013) [35]	Hospital	Ethiopia	Unspecified	6.0	CSME
Kaidonis (2014) [71]	Pooled population from 11 studies	Australia	Types 1 and 2	7.6	DME

*DR* diabetic retinopathy, *DME* diabetic macular edema, *CSME* clinically significant macular edema

In the Diabetic Retinopathy Screening Service for Wales, a high prevalence of DR (56.0 % in type 1 diabetes, 30.3 % in type 2 diabetes) was reported, but the prevalence of DME was not found to be higher than other studies (4.2 % in type 1 diabetes, 1.4 % in type 2 diabetes) [10].

There were a few outliers among the studies that reported exceptionally high prevalence of DME. In Kenya, a population-based study found a prevalence of DME of 33.3 % among participants with diabetes [34], while a Canadian study found DME prevalence to be 15.7 %. It is difficult to ascertain if this abnormally - high observed prevalence is due to genuinely high prevalence in these populations or a difference in methodology. Of note, clinical stereoscopic fundus examination by an ophthalmologist was carried out in both of these studies and factored in the diagnosis of DME whereas most studies relied on non-stereoscopic fundus photographs alone, thus raising the question if prevalence studies using non-stereoscopic fundus photographs may be severely underdiagnosing DME. In patients with newly diagnosed diabetes, observed prevalence of DME was almost non-existent, with studies reporting it to be within 0 to 0.8 % [21, 39]. A Cochrane review of prevalence of DME assessed by optical coherence tomography (OCT) has found a large range of prevalence rates (19–65 %) [72]. Of note, none of the studies included in the review were population-based studies. OCT-detected DME was found to have a great degree of disagreement with the clinical definition of CSME, and not all patients who had macular thickening detected on OCT progressed to have clinical DME, hence its validity as a diagnostic tool in epidemiologic studies is questionable.

### Incidence of DME

Cohort studies that investigated DME incidence are summarized in Table 5. Only studies conducted in the US and Europe investigated DME incidence. The WESDR cohort of patients with type 1 diabetes had the longest follow-up time of 25 years [73]. Interestingly, cumulative incidence of DME and CSME in this cohort seemed to plateau at the 14-year mark (DME 26.1 %, CSME 17.0 %), with the latter 11 years adding minimally to the 25-year cumulative incidence (DME 29 %, CSME 17 %). Data available on DME incidence in type 2 diabetes is limited and inconsistent [50, 52, 59].

**Table 5:** Incidence of diabetic macular edema among diabetic subjects

Author (Year)	Type of study	Location	Type of diabetes	Follow-up time in years	Cumulative incidence, % (95 % CI)	Definition of macular edema
Younis (2003) [46]	Population	United Kingdom	Type 1	6	3.2 (0.8–5.7)	DME
Younis (2003) [50]	Population	United Kingdom	Type 2	6	6.1 (4.4–7.8)	DME
Jones (2012) [52]	Population	United Kingdom	Type 2	10	1.5	DME
Martin-Merino (2012) [47]	Population	United Kingdom	Type 1	9	4.4	DME
			Type 2		3.6	DME
Thomas (2012) [49]	Population	United Kingdom	Type 2	4	1.4	DME
Perol (2012) [213]	Hospital	France	Unspecified	3	0	DME
Romero-Aroca (2011) [77]	Hospital	Spain	Type 1	10	11.07	DME
Salinero-Fort (2013) [59]	Population	Spain	Type 2	4	0.01	DME
Klein (1998) [43]	Population	United States of America	Type 1	14	26.1 (22.6–29.6)	DME
					17.0 (14.1–19.9)	CSME
Klein (2009) [73]	Population	United States of America	Type 1	25	29	DME
					17	CSME
Varma (2010) [54]	Population (Latino)	United States of America	Unspecified	4	5.4 (3.8–7.1)	DME exclusive of CSME
					7.2 (5.2–9.1)	CSME
Leske (2006) [57]	Population	Barbados	Types 1 and 2	9	8.7 (5.4–12.0)	CSME

*DR* diabetic retinopathy, *DME* diabetic macular edema, *CSME* clinically significant macular edema, *CI* confidence interval

### Risk factors for DR and DME

DR and DME share many common risk factors. Incidence-derived risk factors for DR and DME reported in the various cohort studies are summarized in Table 6. The major and established risk factors have been reviewed extensively before [74]. The most pertinent observations will be highlighted again in this review, with updates from the latest literature. Novel risk factors were also reviewed.

**Table 6:** Incidence-derived risk factors for the development of diabetic retinopathy in cohort studies

Risk factor	Author (Year)	Strength of association
Age	Xu (2014) [75]	OR (95 % CI) = 1.00 (0.98–1.02) per year increase
	Ahmed (2012) [210]	HR (95 % CI) = 1.29 (1.07–1.58) per year
	Janghorbani (2003) [212]	HR (95 % CI) = 1.03 (1.006–1.04) per year increase
	Jones (2012) [52]	Compared to 40–70 years, HR (95 % CI) = 1.49 (1.09–2.05) for < 40 years; 1.26 (1.00–1.27) for > 70 years
Gender	Xu (2014) [75]	OR (95 % CI) = 1.32 (0.88–1.96) *reference gender not reported
	Kajiwara (2014) [76]	HR (95 % CI) = 1.85 (1.06–3.24) for female
	Ahmed (2012) [210]	HR (95 % CI) = 1.08 (0.91–1.29) *reference gender not specified
Smoking	Stratton (2001) [51]	OR (95 % CI) = 0.63 (0.48–0.82) if current smoker
Duration of diabetes	Xu (2014) [75]	OR (95 % CI) = 1.16 (1.10–1.22) per year increase
	Kajiwara (2014) [76]	OR (95 % CI) = 1.13 (1.09–1.17) per year increase
	Romero-Aroca (2011) [77]	OR (95 % CI) = 8.90 (4.83–17.4) for ≤ 15 years vs. > 15 years
	Jones (2012) [52]	Compared to < 10 years, HR (95 % CI) = 1.21 (1.01–1.44) for 10–20 years; 0.93 (0.68–1.26) if ≥ 20 years
	Thomas (2012) [49]	Compared to < 5 years, HR (95 % CI) = 1.29 (1.23–1.34) for 5–9 years; 1.68 (1.59–1.77) for 10 years
	Salinero-Fort (2013) [59]	Compared to < 6 years, HR (95 % CI) = 1.22 (0.88–1.70) for 7–14 years; 1.64 (1.05–2.57) for 15–22 years; 2.00 (1.18–3.39) for 22 years
HbA1C	Xu (2014) [75]	OR (95 % CI) = 1.73 (1.35–2.21) per 1 % increase
	Jin (2014) [98]	OR (95 % CI) = 1.12 (1.01–1.24) per 1 % increase
	Tam (2009) [60]	OR (95 % CI) = 1.57 (1.23–2.00) per 1 % increase
	Kajiwara (2014) [76]	OR (95 % CI) = 1.21 (1.08–1.36) per 1 % increase
	Stratton (2001) [51]	Compared to HbA1C < 6.2 %, OR (95 % CI) = 1.4 (1.1–1.8) for 6.2–7.4 %; 2.5 (2.0–3.2) for > 7.4 %
	Romero-Aroca (2011) [77]	OR (95 % CI) = 4.01 (1.91–8.39) if > 7.0 % vs. ≤ 7.0 %
	Tudor (1998) [53]	OR (95 % CI) = 1.50 (0.96–2.36) per 2 % increase
	Kajiwara (2014) [76]	HR (95 % CI) = 1.33 (1.18–1.51) per 1 % increase
	Janghorbani (2003) [212]	HR (95 % CI) = 1.08 (1.007–1.15) per 1 % increase
	Salinero-Fort (2013) [59]	Compared to HbA1C < 7 % HR (95 % CI) = 1.39 (1.01–1.92) for 7–8 %; 1.90 (1.30–2.77) for > 8 %
	Henricsson (2003) [111]	HR (95 % CI) = 1.7 (1.43–1.93) per 1 % increase
Use of insulin/diabetes treatment	Tudor (1998) [53]	OR (95 % CI) = 2.00 (0.75–5.35) if on oral treatment vs. no medications
		OR (95 % CI) = 9.30 (2.69–32.16) if on insulin vs. no medications
	Jones (2012) [52]	Compared to diet control only, HR (95 % CI) = 1.77 (1.44–2.17) if oral hypoglycemics only
		HR (95 % CI) = 2.17 (1.68–2.81) if using insulin
	Thomas (2012) [49]	Compared to diet control only, HR (95 % CI) = 1.41 (1.36–1.47) if oral hypoglycemics only
		HR (95 % CI) = 2.03 (1.89–2.18) if using insulin
Blood pressure	Jin (2014) [98]	OR (95 % CI) = 1.80 (1.14–2.86) if SBP > 140 mmHg and/or DBP > 90 mmHg
	Kajiwara (2014) [76]	OR (95 % CI) = 1.02 (1.01–1.03) per mmHg increase in SBP
	Stratton (2001) [51]	Compared to < 125 mmHg, OR (95 % CI) = 1.5 (1.2–2.6) for SBP was 125–139 mmHg; 2.8 (2.2–3.5) if SBP was ≥ 140 mmHg
	Romero-Aroca (2011) [77]	OR (95 % CI) = 3.31 (1.62–6.75) if SBP > 140 mmHg and/or DBP > 90 mmHg
	Tudor (1998) [53]	OR (95 % CI) = 1.81 (1.02–3.20) per 20 mmHg increase in SBP
	Kajiwara (2014) [76]	HR (95 % CI) = 1.01 (0.99–1.03) per mmHg increase in SBP
	Jones (2012) [52]	HR (95 % CI) = 0.72 (0.64–0.81) if on anti-hypertensive medications
Obesity	Kajiwara (2014) [76]	OR (95 % CI) = 1.07 (1.01–1.13) per kg/m^2^ increase in BMI
	Kajiwara (2014) [76]	HR (95 % CI) = 1.16 (1.06–1.26) per kg/m^2^ increase in BMI
	Henricsson (2003) [111]	HR (95 % CI) = 1.11 (1.04–1.18) per kg/m^2^ increase in BMI
Nephropathy	Xu (2014) [75]	OR (95 % CI) = 1.01 (1.002–1.022) per mmol/L increase in serum creatinine concentration
Axial Length	Xu (2014) [75]	OR (95 % CI) = 0.48 (0.33–0.71) per mm increase
Cerebrospinal fluid pressure	Xu (2014) [75]	OR (95 % CI) = 1.10 (1.01–1.21) per mmHg increase
Fasting blood glucose	Janghorbani (2003) [212]	HR (95 % CI) = 1.003 (1.0003–1.005) per mg/dL increase in fasting blood glucose
Cholesterol	Salinero-Fort (2013) [59]	Compared to < 100 mg/dL, HR (95 % CI) = 0.87 (0.65–1.16) for LDL 100–190 mg/dL; 7.91 (3.39–18.47) for LDL > 190 mg/dL
Aspirin use	Salinero-Fort (2013) [59]	HR (95 % CI) = 1.65 (1.22–2.24) if patient takes aspirin
Risk factors for DME Incidence		
Risk factor	Author (Year)	Strength of association
Duration of diabetes	Romero-Aroca (2011) [77]	OR (95 % CI) = 8.921 (4.321–26.773) if > 15 years of diabetes duration
HbA1c	Romero-Aroca (2011) [77]	OR (95 % CI) = 3.121 (1.823–10.332) if HbA1c is > 7.0 %
Blood pressure	Klein (2009) [73]	HR (95 % CI) = 1.17 (1.10–1.25) per 1 % increase
	Romero-Aroca (2011) [77]	OR (95 % CI) = 3.115 (0.907–10.70) if SBP > 140 mmHg and/or DBP > 90 mmHg
	Klein (2009) [73]	HR (95 % CI) = 1.15 (1.04–1.26) for every 10 mmHg increase in SBP
Nephropathy	Romero-Aroca (2011) [77]	OR (95 % CI) = 6.774 (3.442–18.236) if protein excretion > 200 μg/min or > 300 μg/mg of albumin: creatinine ratio
	Klein (2009) [73]	HR (95 % CI) = 1.43 (0.99–2.08) if urine protein concentration ≥ 30 mg/dL
Cholesterol	Romero-Aroca (2011) [77]	OR (95 % CI) = 4.125 (1.125–15.857) if Total cholesterol/HDL-cholesterol ratio is > 3.5 in men and > 3.0 in women

*OR* odds ratio, *HR* hazard ratios, *CI* confidence interval, *LDL* low density lipoprotein, *HDL* high density lipoprotein, *SBP* systolic blood pressure, *DBP* diastolic blood pressure

#### Non-modifiable risk factors

### Duration of diabetes

Cohort studies with the longest follow-up times found that almost all patients with type 1 diabetes develop some degree of retinopathy if duration of disease exposure is long enough [44, 45]. This relationship is not as clear in cohort studies on type 2 diabetes, probably due to the competing risk of mortality in patients with type 2 diabetes, who are older and may have more age-related comorbidities. Nevertheless, many studies, both in type 1 and type 2 diabetes [49, 52, 59, 75–77], found disease duration to be a significant risk factor for DR, and this is independent of adequacy of glycemic control.

### Puberty and pregnancy

Puberty is a well-known risk factor for DR in type 1 diabetes. Pre-pubertal years of diabetes exposure contributes to added risk of DR [78, 79], but it seems that it is disease exposure during puberty itself, when the body is undergoing rapid development and maturation, that has the greater impact on the risk of DR. In Finland, the FinnDiane Study Group found that onset of diabetes during pubertal or post-pubertal age increases risk of developing severe retinopathy requiring laser treatment when compared to patients with pre-pubertal onset of diabetes [80]. This was particularly significant among the male participants. Biological pathways that may contribute to this phenomenon include the transforming growth factor beta (TGF-β) signaling pathway, which is an important mediator of renal microvascular damage [81]. Androgens promote and accelerate TGF-β transcriptional activity, which can explain the male preponderance. However, evidence of activation of similar pathways in retinal vessels is lacking.

DR and DME can progress rapidly during pregnancy, especially in patients with type 1 diabetes. A recent study found progression of DR in pregnancy to be almost 3 times as likely to occur in mothers with type 1 diabetes as mothers with type 2 diabetes (31.3 vs. 11.7 %, p = 0.001) [82]. This progression is often transient and accompanied by rapid regression of DR in the postpartum period. At the end of 6.5 years of follow-up on average, prevalence and severity of retinopathy was comparable between women with pregnancies and women without pregnancies [83]. Possible mechanisms behind the progression of DR in pregnancy include both hormonal and immune theories [84, 85].

#### Modifiable risk factors

### Hyperglycemia

Hyperglycemia is one of the most important risk factors for DR and DME. A meta-analysis of three large population-based studies found a graded relationship between the level of glycemia and frequency of retinopathy signs [86]. The United Kingdom Prospective Diabetes Study (UKPDS) and the Diabetes Control and Complications Trial (DCCT) provided strong evidence that tight control of glycemia (HbA1c <7 %) reduces the risk of development and progression of DR in both type 1 and type 2 diabetes [87]. The DCCT showed that intensive glycemic control reduced the incidence of retinopathy by 76 % and progression from early to advanced retinopathy by 54 % [88]. This highlights that strict glycemic control is much more effective in preventing or delaying the onset of DR in patients with diabetes without DR, rather than limiting the severity of DR after it has occurred. In the case of DME, intensive glycemic control was associated with 46 % reduction in the incidence of DME at the end of the trial and a 58 % reduction 4 years later compared with those in the conventional group [89]. The burden of primary prevention of DR and DME hence falls heavily on primary care physicians, who are in the best position to achieve good glycemic control in patients who have not developed complications. In everyday clinical care however, it is difficult to replicate the intensity of glycemic control seen in these studies that were achieved under trial conditions. From the findings reported by the DCCT, intensive glycemic control actually increases risk of progression of existing DR in the first year of treatment [90]. However, this should not deter achieving tight glycemic control in patients with existing DR, as the long-term progression risk reduction outweighs that of the increased risk in the first year alone.

Glycemic control should be achieved early in the disease course and maintained for as long as possible, since its protective effect is sustained even if tight glycemic control is lost. This is the metabolic memory effect observed after the DCCT. Within a year after the end of DCCT, the glycemic control in the conventional group and intensive control group had converged, but the participants in the intensive control group still had lower prevalence of DR and DME than the participants in the conventional control group at 10 years after DCCT [91]. Risk reduction in the intensive control group was 52 % between years 1 to 10 after DCCT, but dwindled to 12 % between years 11 to 18 [92]. This implies that the metabolic memory effect fades with time, but this is confounded by improved glycemic control and risk reduction in the conventional control group since the end of DCCT. Besides implications for clinical treatment, metabolic memory also has implications on methodology of diabetes research, seeing that obtaining mean HbA1c of the entire course of diabetes may be needed to control for the effect of metabolic memory [93].

Apart from the absolute value of glycemia alone, the short-term variability of glycemia, such as spikes in post-prandial glucose, is found to be associated with increased risk of microvascular complications [94]. However, there is insufficient data at this point to conclude that fluctuations in blood sugar levels is a causative factor in microvascular complications considering increased glycemic fluctuation can be due to a multitude of correlated factors that may all contribute to microvascular injury, such as severity of disease or poor compliance.

The benefits of achieving euglycemia should be balanced with the risk of hypoglycemia, especially in the elderly. In both the Action in Diabetes and Vascular Disease (ADVANCE) [95] and Action to Control Cardiovascular Risk in Diabetes (ACCORD) [96] trials, aggressive glycemic control (HbA1c <6.5 %) did not significantly reduce risk of retinopathy development or progression in type 2 diabetes. In ACCORD, it was found that such an aggressive manner of glycemic control may in fact be associated with increased mortality, but it was not ascertained whether this was directly due to metabolic complications of treatment, such as hypoglycemia. Current institution guidelines state that treatment goals of hyperglycemia are to be anywhere between <6.5 to <7.5 % of HbA1c. According to a recently published Cochrane review [97] however, there is no concrete evidence on any specific treatment target. Instead, the authors recommend that clinicians set individualized treatment goals based on age, disease progression, risk of hypoglycemic episodes, and psychological factors of the patient.

### Hypertension

Multiple epidemiologic studies have identified hypertension as a risk factor for DR and DME [51, 53, 76, 77, 98]. In the UKPDS, tight blood pressure control (defined as target blood pressure <150/85 mmHg) in patients with type 2 diabetes reduced the risk of microvascular disease by 37 %, the rate of progression of DR by 34 %, and the risk of deterioration of visual acuity by 47 % [99]. Unlike in the case of hyperglycemia, the protective effect of blood pressure control waned quickly upon stopping intensive control [100]. Anti-hypertensive medications targeting the renin-angiotensin-aldosterone system (RAAS) are now the first line treatment for control of hypertension in patients with nephropathy as it was found that they had additional beneficial effects independent of their absolute hypotensive action. Since retinopathy and diabetic nephropathy are related microvascular complications, clinical trials such as the Diabetic Retinopathy Candesartan Trials (DIRECT) and Renin-Angiotensin System Study (RASS) measured the beneficial effects these classes of anti-hypertensive medications had on DR and DME. Candesartan was found to reduce the incidence of retinopathy by two or more steps in severity on the ETDRS scale by 18 % or by three or more steps by 35 % in type 1 diabetes, and increased regression of retinopathy by 34 % in type 2 diabetes [101, 102]. However, regression only occurred in mild DR, and candesartan had no effect on incidence or progression of DME. In the RASS, enalapril and losartan reduced the risk of retinopathy progression by 65 and 70 %, respectively. Since it was observed that this effect was independent of blood pressure changes across the period of the trial, it was proposed that DR risk reduction was not mediated by an effect on hypertension.

A recently published Cochrane review concluded that intensive blood pressure control had a modest effect in reducing incidence of DR, but does not reduce risk of progression [103]. Insufficient evidence on adverse effects of strict blood pressure control in patients with diabetes made a cost-benefit analysis impossible in the review, and both clinicians and researchers should be aware of this gap in literature. Hence, the overall recommendation is to avoid intensive blood pressure control for the sole purpose of slowing DR progression. Instead, control of hypertension in a patient with diabetes should be focused on preventing or limiting progression of other vascular complications, particularly nephropathy, as well as lowering mortality. There is insufficient evidence for the use of RAS targeting anti-hypertensive medication specifically for preventing or treating retinopathy.

### Dyslipidemia

As outlined in a previous review, the evidence for dyslipidemia as a risk factor for DR are inconsistent, and no single lipid measure had been consistently found to be associated with DR or DME [74]. In recent cohort studies, only the Madrid Diabetes Study found an association between low density lipoprotein (LDL) cholesterol and incidence of DR [59]. Moreover, a meta-analysis found that there was a dose-dependent relationship of statin use with increasing risk of diabetes [104]. It was then believed that statins might have effects on glucose homeostasis, such as decreasing insulin production or increasing insulin resistance, or both [105]. Therefore, while the use of statins is first-line treatment for dyslipidemia in the prevention of cardiovascular events in patients with diabetes, the evidence for intensive control by statins for the purposes of treating DR and DME are lacking.

Fenofibrate, a peroxisome proliferator-activated receptor alpha (PPARα) agonist, has gathered interest on its effects on DR and DME. In an ancillary study of the Fenofibrate Intervention and Event-lowering in Diabetes (FIELD) cohort, participants treated with fenofibrate had a 31 % reduced risk of requiring laser treatment for PDR or DME, compared to placebo [106]. However, 2-step progression of retinopathy did not differ significantly between the fenofibrate and placebo group, except for the subgroup with pre-existing DR. In this subgroup, risk of 2-step progression was almost a fifth of that compared to placebo. Moreover, in a more recent trial by the ACCORD group, adjunct fenofibrate with simvastatin compared to simvastatin alone reduced the rate of progression of DR (6.5 vs. 10.2 %, respectively) by at least 3 steps at 4 years [107]. Fenofibrate treatment may also have beneficial effects on DME, as it was found to have a moderate effect in decreasing macular volume in patients with DME [108]. The sample size of this study however, was relatively small, and more studies are required to study this association. Given the current evidence, it is found that patients with DR benefit most from fibrate therapy if they have hypertriglyceridemia and low serum high density lipoprotein (HDL)-cholesterol, and hence treatment can be justified in this subset of patients, with the hopes of slowing progression to PDR. However, generalization of fibrate treatment to all patients with diabetes at risk of DR is not recommended without stronger evidence [109].

### Obesity

The effect obesity has on DR has been relatively well-studied but with inconclusive and conflicting findings [110]. It may be possible that obesity has differing impacts on DR in type 1 diabetes as compared to type 2 diabetes. In the Diabetic Incidence Study in Sweden involving predominantly participants with type 1 diabetes, it was found that risk of developing DR increased by 1.11 (95 % confidence interval (CI) 1.04–1.18) times per kg/m^2^ increase in Body Mass Index (BMI) after 10 years of follow-up [111]. In the EURODIAB Prospective Complications Study, also involving patients with type 1 diabetes, larger waist to hip ratio was associated with incidence of DR after more than 7 years of follow-up [112].

In contrast, many studies in type 2 diabetes, performed primarily in Asia, found an inverse relationship between obesity and DR. In a cross-sectional study of the Shanghai Diabetes Registry Database, participants who were overweight had reduced risk of DR and VTDR [113]. A similar study on the multi-ethnic population in Singapore found the same risk reduction in obese patients for DR, VTDR and CSME [114].

The exact mechanisms underlying this discrepancy between type 1 and type 2 diabetes are not well understood. It was postulated that unintentional weight loss is a sign of advanced and severe type 2 diabetes, hence the observation of non-obese patients with type 2 diabetes being at higher risk of DR. In contrast, obesity and metabolic syndrome do not contribute to the etiology of type 1 diabetes, which is autoimmune in nature, and obese patients with type 1 diabetes may simply have more difficulties achieving good glycemic control. It should be noted that there are no prospective population-based studies in Asia on DR incidence, and the protective effect of obesity in Asians with type 2 diabetesis yet to be confirmed by a cohort study.

Closely related to obesity is the study of obstructive sleep apnea (OSA) as a potential risk factor for DR and DME. A cross-sectional study in patients with type 2 diabetes found that OSA was associated with DR severity, but not DME [115]. A separate study on patients with CSME found high prevalence of sleep-disordered breathing in these patients, but severity of sleep-disordered breathing was not correlated with severity of DR or DME in this study [116]. However, the sample sizes of these studies were too small to draw any concrete conclusions.

Bariatric surgery is a highly effective treatment for morbid obesity that achieves glycemic control of diabetes rapidly. However, much like how intensive glucose control with medications or insulin increases risk of DR progression in the short-run, this rapid improvement in glycemic control post-bariatric surgery has been associated with progression of DR. Most studies presented in this area are case series, and a recent meta-analysis of these studies found that patients with pre-existing DR are 2.77 times (95 % CI 1.10–6.99) more likely to have adverse outcomes in DR post-operatively than patients without pre-existing DR [117]. As mentioned earlier, increased risk of progression with intensive glycemic control occurred only in the first year of follow-up, with subsequent risk reduction with longer-term control [90]. It remains to be seen if this is the case with bariatric surgery as well, as no studies had sufficient follow-up time to determine if bariatric surgery has long-term benefits on DR.

#### Novel risk factors

### Inflammation

Retinal and vitreous inflammation was observed in subjects with diabetes, both in animal models and human studies. The role of inflammation in DR and DME is therefore an area of extensive study, and has been reviewed previously [118]. As pointed out in the review however, current data suggests systemic inflammation cannot account for the characteristic lesions seen in DR and DME. Many conditions can lead to systemic inflammation (e.g. sepsis, autoimmune disease), but DR-like lesions and DME are not seen in these diseases. Hence, it seems that the local retinal inflammation seen in subjects with diabetes is not related to systemic inflammation. This challenges the validity of investigating systemic inflammatory markers such as serum C-reactive protein (CRP), interleukin-6 (IL-6) and tumor necrosis factor-α (TNF-α) as risk factors for DR or DME. Indeed, inconsistencies in the association between systemic inflammatory markers and risk of DR and DME exist in the current literature. The EURODIAB Prospective Complications Study found an association between CRP, IL-6, TNF-α and presence of DR in subjects with type 1 diabetes via a cross-sectional study [119]. Other cross-sectional studies found no such association. The Multi-ethnic Study of Atherosclerosis did not find an association between CRP and DR or VTDR (which includes DME), but found an association between fibrinogen, an acute-phase reactant in systemic inflammation, and DR and VTDR [120]. The Singapore Malay Eye Study even found that raised CRP was associated with a lower prevalence of DR [121]. None of the studies found an association between systemic inflammatory markers and DME specifically.

Local retinal inflammation forms the basis of intravenous administration of corticosteroids. The Diabetic Retinopathy Clinical Research Network (DRCR.net) compared intravitreal triamcinolone versus focal/grid laser photocoagulation in patients with DME. The findings showed that the triamcinolone group had better visual acuity at the 4-month interval, but equivalent visual acuity at the 1-year interval. At the 2-year [122] and 3-year interval [123], mean visual acuity was better in the photocoagulation than the triamcinolone groups. Hence, corticosteroid treatment for DME is effective, but the effect is transient. Clinicians also have to be cautious with adverse effects such as elevated intraocular pressure and cataract formation.

Vascular endothelial growth factor (VEGF) is a key modulator of angiogenesis and vascular permeability, and is upregulated by inflammatory cytokines [124]. Anti-VEGF agents have been used successfully for the treatment of both PDR and DME [125, 126]. Ranibizumab, an anti-VEGF agent, was more effective than laser therapy in restoring vision for DME [127], although just like with corticosteroids, ranibizumab is associated with elevations in intraocular pressure [128]. In recent reports, the DRCR.net compared outcomes in DME treated by aflibercept, bevacizumab or ranibizumab, and found that aflibercept provided superior visual recovery if baseline visual acuity was poorer than 69 ETDRS letters (approximately 6/15 Snellen) when compared to the other anti-VEGF agents, but there was no significant difference between aflibercept and the other anti-VEGF agents if baseline visual acuity was better than 69 letters [129].

Anti-VEGF agents appear superior to corticosteroids in terms of efficacy. DRCR.net compared ranibizumab and concurrent photocoagulation against triamcinolone with photocoagulation in patients with DME, and found that ranibizumab achieved better visual outcome at 1-year follow-up than triamcinolone, except in a subset of patients with pseudophakic eyes [130]. In this subset of participants, triamcinolone achieved comparable visual outcome when compared with ranibizumab, possibly because of the removed effect of steroid-induced cataract formation in pseudophakic eyes. Consistent results were obtained at 2-year follow-up [131].

### Metabolic hormones

Hormones involved in metabolism have been hypothesized to play key roles in the pathogenesis of microvascular complications in diabetes, due to their roles in both metabolic and inflammatory pathways [132]. In particular, leptin and adiponectin, which are actively secreted by adipocytes to regulate energy balance in the body, have been implicated as potential risk factors.

Leptin may play a role in inciting inflammation. Leptin was found to cause upregulation of VEGF in retinal pericytes [133], hence stimulating angiogenesis in the ischemic retina [134], and possibly contributing to the neovascularization seen in PDR. Elevated serum and vitreous leptin was observed in patients with diabetes, and vitreous leptin was especially elevated in patients with PDR [135]. However, cross-sectional studies could not find an association between elevated serum leptin and DR [136, 137], though it should be noted that the sample sizes of these studies were relatively small and they may be underpowered.

Adiponectin has been found to induce dilation of retinal arterioles via upregulation of endothelial cell nitric oxide production, in animal studies [138]. Studies by the same group in human subjects with mild DR found that serum adiponectin was positively correlated with retinal blood flow velocity and negatively correlated with retinal arterial resistance [139]. Hence, adiponectin may have a role in countering ischemia by promoting reperfusion in the ischemic retina. *In vitro* studies also found that it downregulates VEGF and thus may have anti-angiogenic properties [140]. Large population-based cross-sectional studies found that elevated serum adiponectin in patients with DR correlated with severity of DR when compared to patients without DR [141, 142]. However, there are inconsistencies in literature, with one study finding decreased serum adiponectin in participants with PDR [143]. Given that basic science suggests adiponectin as mainly protective against the development of microvascular complications, the observation that serum adiponectin is elevated in patients with severe DR appears contradictory. It may be that upregulation of adiponectin secretion can be attributed to a natural response that ameliorates the effects of severe microvascular disease, but prospective cohort studies are needed to establish the temporal link between adiponectin levels and the development and progression of DR. Overall, it appears research in adiponectin has produced more promising and consistent results than leptin. The association between these hormones and DME has yet to be studied.

### Oxidative stress

Oxidative stress is the accumulation of free radicals in the form of reactive oxygen species (ROS). Highly efficient physiological mechanisms consisting of endogenous free radical scavengers usually keep oxidative stress low. However, under pathological conditions, ROS production may be increased such that the defensive mechanisms are overwhelmed, or the protective mechanisms themselves may be impaired, or both [144]. Oxidative stress has been linked to the histopathological changes of DR, such as retinal basement membrane thickening [145] and capillary cell loss [146]. Increased ROS and decreased antioxidant potential has also been found in patients with diabetes, especially if they have DR [147]. The effects of oxidative stress are observed early in the course of diabetes, and its effects on microvasculature persist even if hyperglycemia is subsequently corrected. Hence, oxidative stress is likely to be the mechanism behind the “metabolic memory” effect mentioned earlier, where sustained periods of hyperglycemia early in the disease course has long-lasting effects on future microvascular complications [148].

Multiple biochemical pathways involved in DR pathogenesis are linked to oxidative stress. The accumulation of advanced glycation end products (AGE) in retinal pericytes upregulates cellular expression of its receptor (RAGE). AGE-RAGE overexpression produces ROS, activating apoptotic pathways to cause pericyte loss, seen in early DR [149] The polyol pathway is augmented in hyperglycemic conditions, resulting in overconsumption of NADPH, reducing its availability for formation of the key endogenous antioxidant glutathione [150]. ROS has also been found to increase the activity of protein kinase C (PKC), a family of serine-threonine kinases that cause vascular dysfunction by increasing permeability, altering blood flow, and stimulating neovascularization. Vascular dysfunction and neovascularization is potentiated further as PKC induces VEGF [144]. Due to how multiple pathways activate and can be activated by oxidative stress, therapeutic strategies targeting any single pathway is unlikely to be effective, as shown in the multiple randomized-controlled trials [151–153]. Research has since focused on mitochondrial dysfunction as the main upstream source of oxidative stress, but whether research in this area will yield novel treatment strategies remains to be seen [148].

From an epidemiologic standpoint, given the importance of oxidative stress in the pathogenesis of DR, reliable and accessible markers of oxidative stress are valuable measures of disease severity and prognosis. To date, most studies relating oxidative stress to DR involve *in vitro* and animal studies, and oxidative stress markers have not been investigated in large epidemiologic studies. Small cross-sectional studies have consistently found elevated markers of oxidative stress such as lipid peroxide (LPO) and malondialdehyde in both vitreous and serum of human subjects with DR [154, 155]. In particular, serum LPO was found to correlate highly with vitreous LPO, and that LPO correlated well with key disease mediators such as VEGF, suggesting that serum LPO may be a suitable proxy measure of DR severity [154]. More studies will be needed to confirm this association.

### Vitamin D

On top of its well-known effects on calcium metabolism, Vitamin D has anti-angiogenic and anti-inflammatory effects that have implicated Vitamin D deficiency in the pathogenesis of various types of pathology, such as malignancy, autoimmune disease, cardiovascular disease and diabetes [156].

It is thus intuitive that Vitamin D has a protective effect on DR and DME, since anti-angiogenesis may slow progression to PDR and anti-inflammatory properties may counteract development of both DR and DME. Calcitriol, or 1,25-dihydroxycholecalciferol, is the metabolically active form of Vitamin D, and has been found to be a potent inhibitor of retinal neovascularization *in vitro* [157], possibly through suppressing TGF-β and VEGF levels [158]. Epidemiologic studies have found vitamin D deficiency to be associated with increased prevalence and severity of diabetic retinopathy, in both type 1 [159, 160] and type 2 diabetes [161–163]. However, all these studies are cross-sectional. No data is available on how Vitamin D influences prevalence of DME.

### Genetic factors

As highlighted earlier in this review, certain trends in DR prevalence and incidence cannot be explained by environmental or socioeconomic factors, such as the abnormally high prevalence of DR in rural China, or the large proportion of VTDR in the Middle East. Some patients appear predisposed to severe DR even with adequate risk factor control, while others avoided DR despite poor control and long diabetes duration [164]. Familial aggregation studies and clinical trials including the DCCT have demonstrated a heritable tendency for severe retinopathy in type 1 and type 2 diabetes, independent of shared risk factors [165–168]. Hence, the hypothesis of differential genetic susceptibility to DR has drawn interest. The list of polymorphisms reviewed here is not exhaustive, but focuses on genes affecting the biological pathways mentioned earlier in the review.

Polymorphisms in the adipose most abundant gene transcript-1 (apM-1) gene on chromosome 1q21.3-q23 that codes for adiponectin have been detected to influence serum adiponectin levels and risk of DR [142]. Participants with type 2 diabetes heterozygous for the Tyr111His polymorphism at exon 3 (Tyr/His) had significantly higher serum adiponectin levels than participants who were homozygous for Tyr111His (Tyr/Tyr), but this had no statistically significant effect on the risk of DR. Participants with type 2 diabetes who had the mutant +45TG allele at the Gly15Gly polymorphism had no observable differences in serum adiponectin levels when compared to participants with the wild type +45TT allele, but they had a significantly lower risk of DR. It was unclear why the reduced risk of DR in this study appeared independent of serum adiponectin levels. Multiple VEGF polymorphisms have been investigated for their link to DR. The -2578C/A, +936C/T and -460 T/C polymorphisms of VEGF have been associated with DR in Asians by meta-analysis of cross-sectional studies [169, 170]; The C-634G polymorphism was linked to risk of DME. The CC genotype of this polymorphism was associated with the presence of DME, but was also associated with better treatment response to bevacizumab when compared to the CG and GG genotypes [171]. Recently, single nucleotide polymorphisms in the VEGF-C gene have been associated with DR and DME in both type 1 and type 2 diabetes [172].

Aldose reductase is the rate-limiting enzyme in the polyol pathway that contributes to oxidative stress in patients with diabetes. The C(−106)T polymorphism was found on meta-analysis to be associated with risk of DR in type 1, but not type 2 diabetes [173]. Genes coding for enzymes in antioxidant pathways such as catalase, superoxide dismutase and glutathione peroxidase are downregulated in patients with DR compared to patients with diabetes but without DR, but it is unknown if certain polymorphisms predispose to this observation [174]. Vitamin D receptor gene polymorphisms may also predispose to DR. T to C substitution at the Taq I site of the Vitamin D receptor gene [175], and T to C substitution at the start codon FokI [176], was associated with severe DR in patients with type 1 diabetes.

A few genome-wide studies have identified novel gene loci associated with DR [177–180]. Association of novel genes related to vascular endothelium proliferation and capillary permeability, such as PLXDC2 and ARHGAP22, imply that our understanding of angiogenic and inflammatory pathways is still incomplete [178]. Interestingly, polymorphism of RP1-90 L14.1, a long intergenic non-coding RNA gene adjacent to CEP162 was found to be associated with susceptibility to DR [180]. Since CEP162 is a key protein in cell ciliogenesis [181], it raises the question if dysregulation of ciliary assembly plays a role in DR pathogenesis.

### Epidemiology of diabetes-related vision loss

While treatment options such as pan-retinal laser photocoagulation can largely control neovascularization and prevent blindness, these treatments cannot restore vision, and in fact have vision-impairing effects of their own. Intravitreal agents such as anti-vascular endothelial growth factor (VEGF) agents do not fully restore vision in all patients, and require frequent and costly doses for effective treatment. Vision loss from DR or DME is hence a significant healthcare burden [1].

A recent systematic review estimated that in 2010, 3.63 million people worldwide suffer from moderate and severe vision loss due to DR and its related sequelae, defined as visual acuity in the better eye being worse than Snellen 6/18 but at least 3/60. An estimated 850 thousand more people suffer from DR-related blindness, defined as visual acuity worse than 3/60 in the better eye [2]. Prevalence of vision impairment and blindness due to DR was found to be on the uptrend, even though total prevalence of vision impairment and blindness was decreasing. Findings from reviews of cross-sectional studies in Europe [182], South-East Asia and Oceania [183], consistently found DR to be the fifth most common cause of moderate and severe vision loss and blindness, behind causes such as uncorrected refractive error, cataracts, macular degeneration and glaucoma. In Africa, DR is the sixth most common cause of visual impairment and blindness, behind the above-listed conditions and trachoma [184]. In the USA, the WESDR investigated visual impairment in patients with type 1 diabetes, and found that 25-year cumulative incidence of visual impairment (defined as poorer than 6/12 best-corrected visual acuity in the better eye) and severe visual impairment (defined as poorer than 6/60 best-corrected visual acuity in the better eye) to be 13 and 3 %, respectively [185].

Recent data in Leeds, UK, found that in 2008 to 2010, DR accounted for 6.1–8.3 % of visual impairment certification. Extrapolated to the total population of the metropolitan area in Leeds, this estimates that 30.0 to 43.2 people per million per year will become severely visually impaired due to DR and its sequelae [186]. In Fife, Scotland, between 2000 and 2009, the mean incidence of blindness (defined as above) was 13.8 per million per year for the total population of the county [187]. In the Sankara Nethralaya Diabetic Retinopathy Epidemiology and Molecular Genetics Study (SN-DREAMS) in type 2 diabetes, the prevalence of visual impairment and blindness was 4 and 0.1 %, respectively [188].

### Other eye complications of diabetes

While DR and DME are the most important and well-studied diabetes-related eye complication, many patients with diabetes are at risk of vision loss from other diabetes-related eye conditions that range from mild vision impairment to blindness. Diabetes is associated with early and rapid development of cataracts, and is hence a major cause of visual impairment among patients with diabetes. The Singapore Malay Eye Study (SiMES) found patients with diabetes to be more likely to have cortical and posterior subcapsular cataracts [189]. In the WESDR study and SN-DREAMS study, presence of cataracts were significant factors contributing to visual impairment and blindness in patients with diabetes [185, 188]. Many patients with diabetes require cataract surgery at a relatively younger age. In the WESDR, 10-year cumulative incidence of cataract surgery was 8 % in patients with type 1 diabetes and 25 % in patients with type 2 diabetes [190]. While usually a surgical procedure with good outcomes, cataract surgery is complicated in patients with diabetes as they may develop DME after surgery [191].

Although findings have been inconsistent, diabetes has been found to be a risk factor for developing primary glaucoma in some population-based studies [192]. For instance, SiMES found an association between ocular hypertension and diabetes, but not glaucoma [189]. Neovascular glaucoma, which is both a blinding and painful condition, can also arise from PDR. A recent report found that 7.1 % of patients with PDR requiring vitrectomy developed neovascular glaucoma 1 year after surgery [193]. Epiretinal membranes, which can cause significant visual impairment, were also found to be more common among patients with diabetes that have undergone cataract surgery [189].

### Relationship of DR and DME with diabetes related systemic complications

#### Microvascular complications

Diabetic nephropathy is closely associated to DR and DME, as many of the pathologic processes affecting microvasculature in DR are likely to be causative of diabetic nephropathy as well. In a cross-sectional study in Korea, compared to patients without DR, patients with DR had 2.11 the odds (95 % CI 1.04–4.26) of having overt diabetic nephropathy, defined as protein excretion of more than 300 mg per 24 h or albumin/creatinine ratio greater than 300 μg/mg [194]. Ischemic diabetic retinopathy, as evidenced by capillary non-perfusion found on fundal fluorescein angiogram, was found to be associated with progression of diabetic nephropathy. Patients with more than or equal to 10 optic disc areas of capillary non-perfusion had 6.64 times the risk of progression of nephropathy [195]. Increasing severity of DR was associated with increasing severity of chronic kidney disease and decreased estimated glomerular filtration rate [196]. In a 15-year follow-up study, development of overt nephropathy (defined as above) was found to be associated with the development of DME [197]. Few studies related the development of neuropathy with DR. However, the SN-DREAMS found an association between neuropathy and visual-impairment in patients with diabetes [188].

### Macrovascular complications

The strength of association between DR and macrovascular complications, such as cardiovascular disease is just as strong as in nephropathy [8]. In the Chennai Urban Rural Epidemiology Study, prevalence of coronary heart disease was higher among patients with DR as compared to those without DR [198]. An eight-year cohort study in Japan found that patients who developed signs of mild DR were already at higher risk of coronary heart disease or stroke [9]. Factoring presence of DR in the assessment of patients with diabetes also improved risk assessment of silent myocardial infarcts [199]. Presence of DR was also associated with mortality from cardiovascular disease, especially if there is concomitant nephropathy [200]. Literature relating DR with peripheral vascular disease is sparse, but a recent cross-sectional study in China found an association between presence of PDR with lower ankle-brachial index and lower toe-brachial index [201].

## Conclusions

As this review shows, the epidemiology of DR has been extensively studied. The use of a common grading system, the ETDRS severity scale and its modifications, has facilitated standardized diagnosis and severity classification of DR in multiple epidemiologic studies, allowing comparisons of prevalence, incidence, progression and regression of DR. Review of literature published within the past five years consistently found higher DR prevalence in Western countries compared to Middle-East and Asian countries. Notable exceptions include Saudi Arabia and Singapore, two of the most affluent countries in Asia, where DR prevalence is comparable to that observed in the US and UK.

Given the increasing affluence of developing economies such as China and India, the healthcare burden of DR can be expected to be on the uptrend in the decades ahead. More recently, cross-sectional studies from developing countries are being published. Understandably, the sample sizes of these studies tend to be small, and few are population-based. However, it is clear that while people in developing countries are at lower risk of developing diabetes, they have an equivalent if not higher risk of developing DR upon onset of diabetes. While traditional causes of visual impairment and blindness in developing countries such as cataracts and trachoma are declining, the prevalence of DR is growing. Gaps in the literature on the epidemiology of DR include the lack of population-based cohort studies investigating the incidence, progression, and regression in Asian and developing-world populations.

In contrast to DR, the epidemiology of DME is much less well studied. Existing studies are split between the use of two diagnostic criteria, one for DME and the other for CSME. Since the CSME criteria are substantially stricter than the DME criteria, direct comparisons between these studies cannot be made. The lack of a severity scale also precludes the study of progression and regression of DME. The diagnosis of DME itself is more challenging than DR. While DR can be diagnosed and classified adequately with the assessment of non-stereoscopic fundus photos, the diagnosis of DME using this same modality is challenging as macular thickening is difficult to assess in non-stereoscopic photographs. There is no consensus on OCT-based severity classification for DME. More research will have to be carried out to overcome these hurdles in diagnosis and classification of DME.

The investigation of risk factors has also revealed interesting considerations both in clinical practice and research. Hyperglycemia remains the most important modifiable risk factor for DR, and intensive glycemic control has been proven to have potent and long-lasting protective effects against development and progression of DR and DME. As the evidence behind hypertension and dyslipidemia as risk factors is weaker than in hyperglycemia, intensive control of hypertension and dyslipidemia should not be sought solely on the basis to prevent onset or progression of DR and DME, but taken in consideration of other complications (e.g. reduction in nephropathy and cardiovascular diseases).

Among novel risk factors, increased serum adiponectin and LPO were found to be associated with greater prevalence of DR. Vitamin D deficiency has also been found to be associated with DR, but more evidence is needed to ascertain efficacy of Vitamin D supplementation in the prevention of DR. These novel risk factors are promising, but the findings that have been made in cross-sectional studies have to be supported by consistent findings in prospective cohort studies. The relationship between these factors and DME is unknown and is worth exploring.

The association between DR and other vascular diseases are important areas of study. DR is strongly associated with nephropathy, which has significant burden on healthcare systems due to the need for renal replacement therapy. The presence of DR is also associated with vascular diseases that are disabling, such as stroke and peripheral vascular disease, or life threatening, such as myocardial infarction. Physicians and ophthalmologists should therefore be aware that patients with DR and DME are receiving appropriate assessment and treatment for these comorbidities.

## Abbreviations

ACCORD: Action to control cardiovascular risk in diabetes
ADVANCE: Action in diabetes and vascular disease
AGE: Advanced glycation end products
apM-1: Adipose most abundant gene transcript-1
CRP: C-reactive protein
CSME: Clinically significant macular edema
DCCT: Diabetes control and complications trial
DCPD1987: Danish cohort of pediatric diabetes 1987
DIRECT: Diabetic retinopathy candesartan trials
DME: Diabetic macular edema
DR: Diabetic retinopathy
DRCR.net: Diabetic retinopathy clinical research network
ETDRS: Early Treatment for diabetic retinopathy study
FIELD: Fenofibrate intervention and event-lowering in diabetes
IL-6: Interleukin-6
OCT: Optical coherence tomography
OSA: Obstructive sleep apnea
PDR: Proliferative diabetic retinopathy
PPARα: Peroxisome proliferator-activated receptor alpha
PKC: Protein kinase C
RAAS: Renin-angiotensin-aldosterone system
RAGE: Receptor of advanced glycation end products
RASS: Renin-Angiotensin System Study
ROS: Reactive oxygen species
SiMES: Singapore Malay Eye Study
SN-DREAMS: Sankara Nethralaya Diabetic Retinopathy Epidemiology and Molecular Genetics Study
TGF-β: Transforming growth factor beta
TNF-α: Tumor necrosis factor-α
UK: United Kingdom
UKPDS: United Kingdom prospective diabetes study
USA: United States of America
VEGF: Vascular endothelial growth factor
VTDR: Vision-threatening diabetic retinopathy
WESDR: Wisconsin epidemiologic study of diabetic retinopathy
